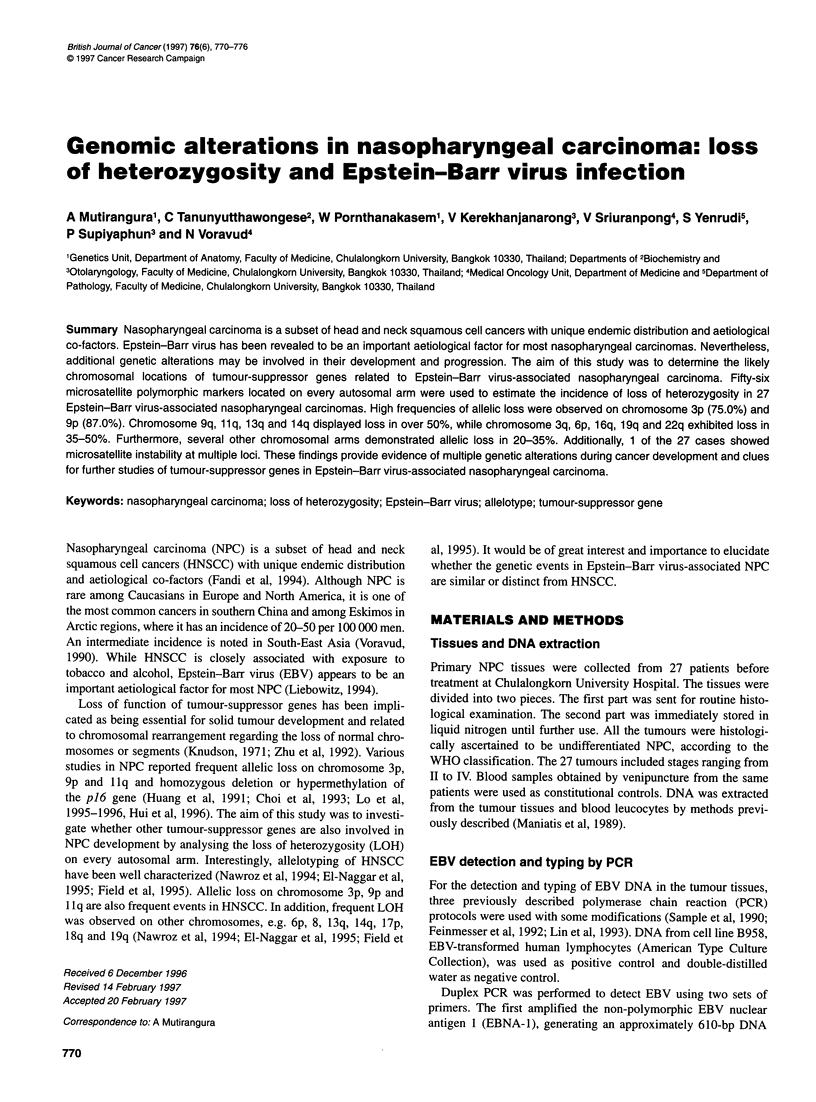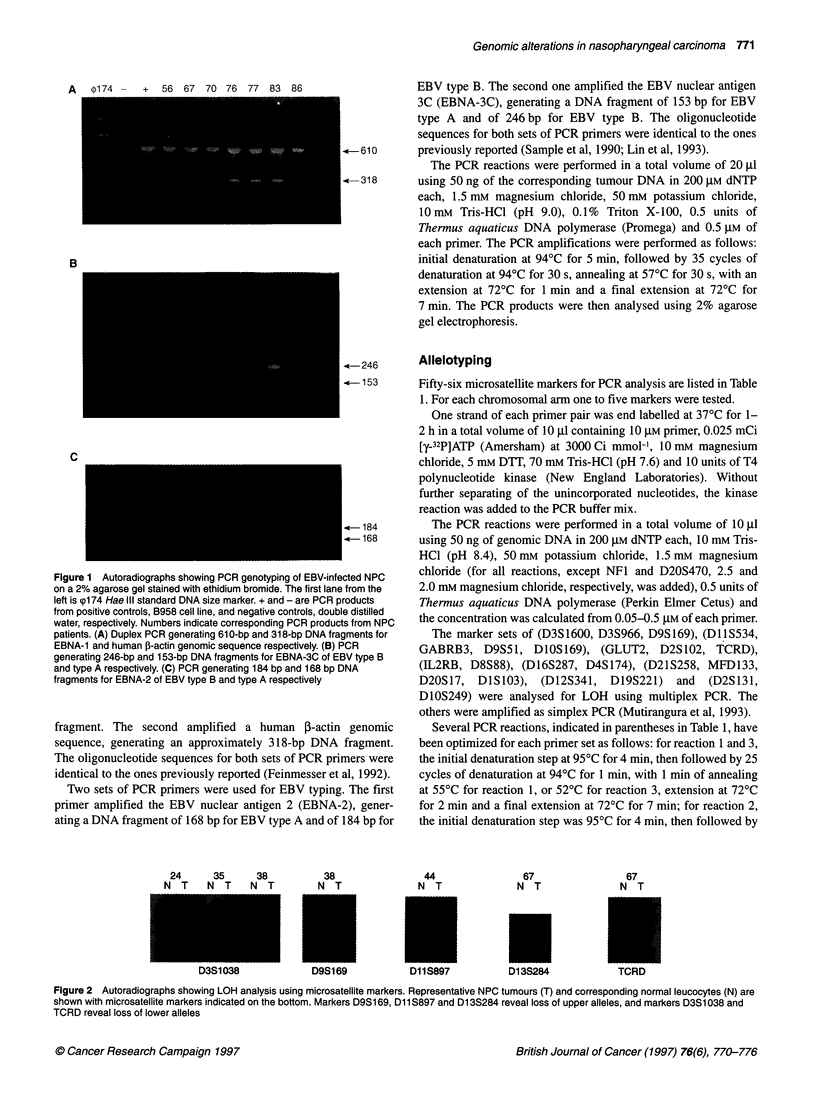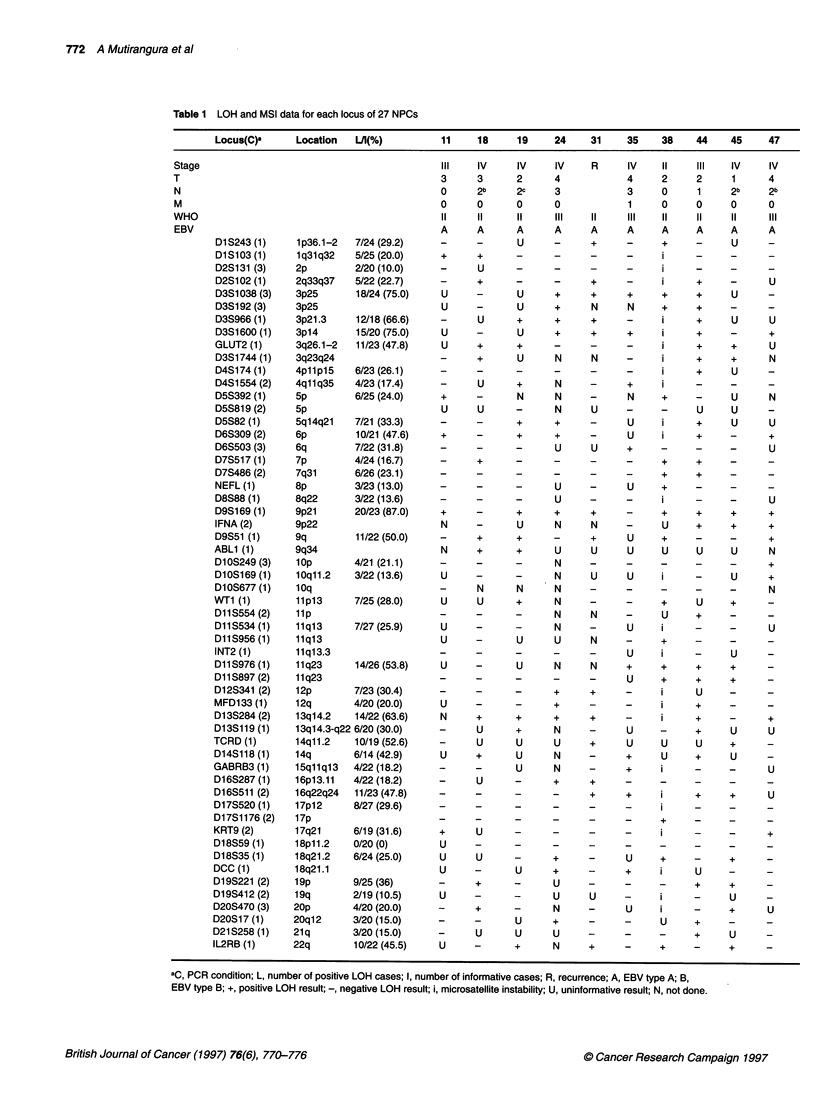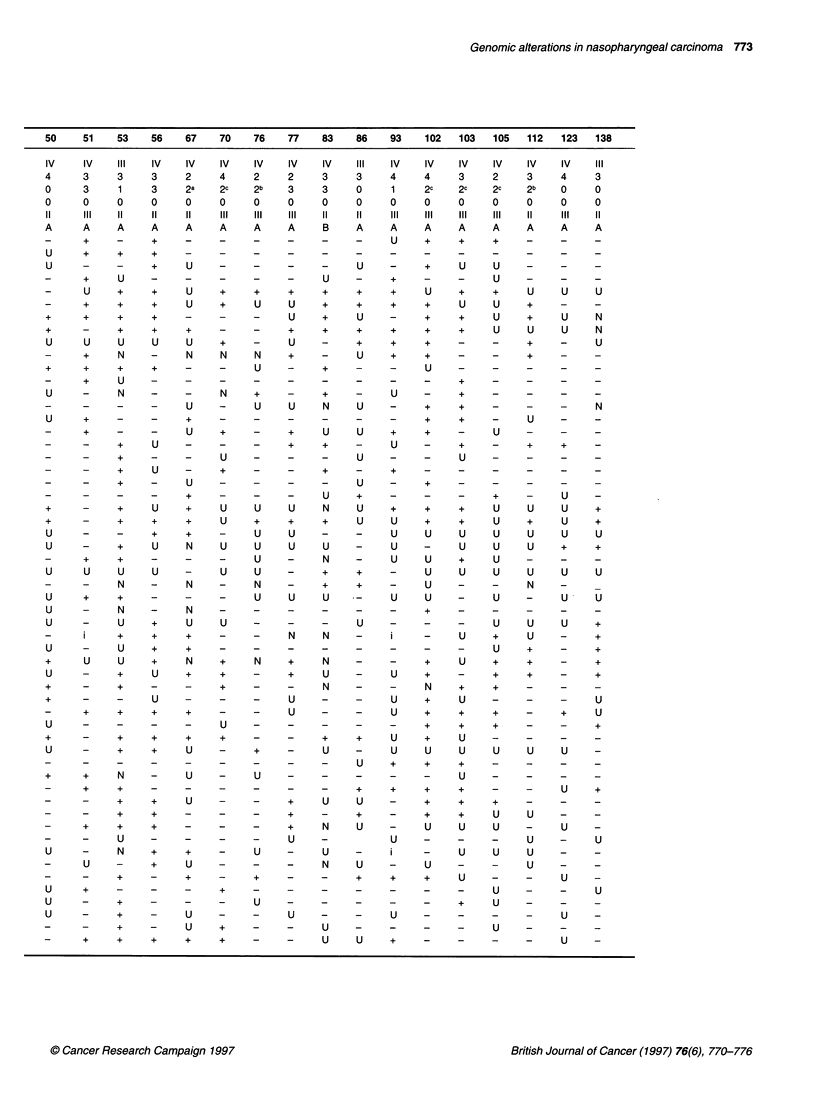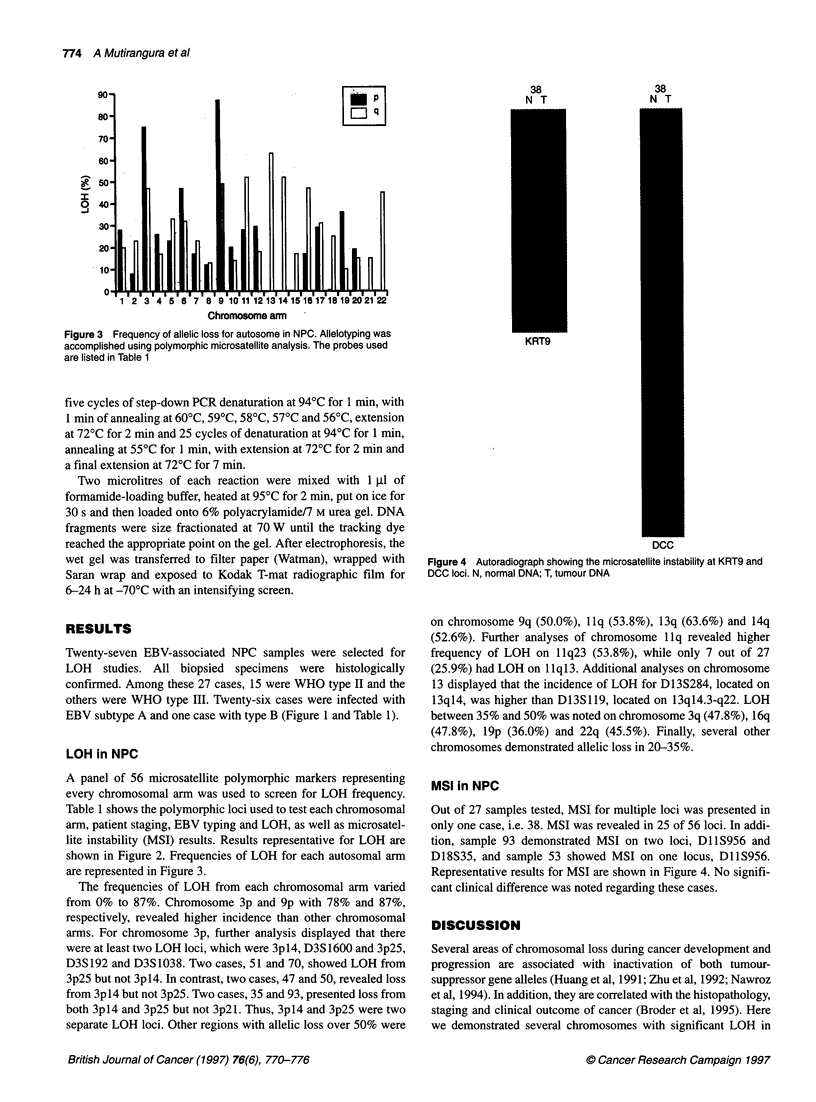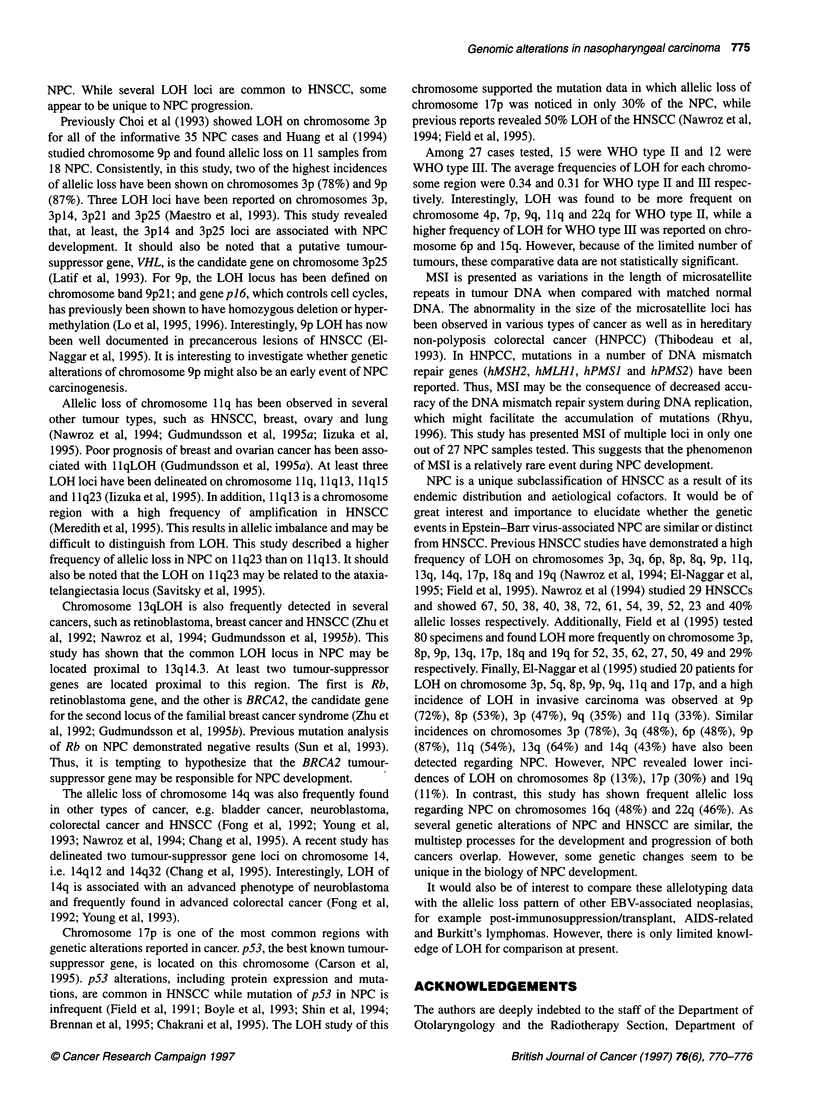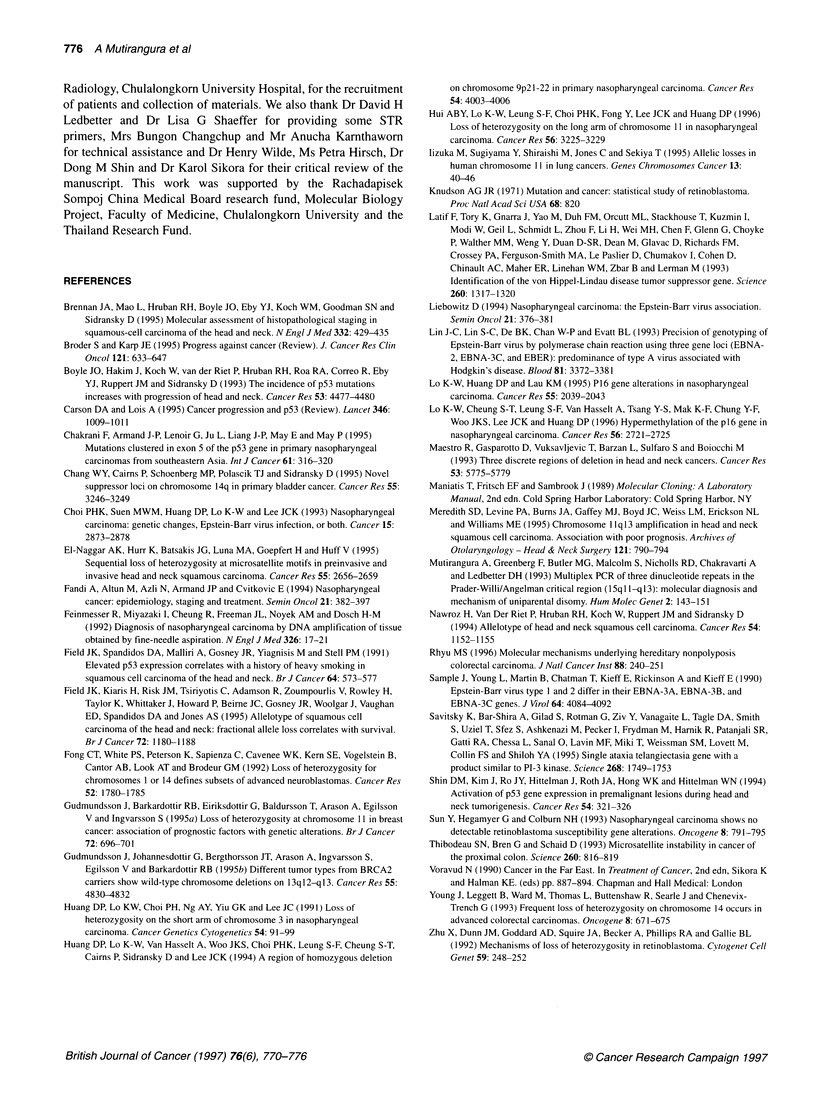# Genomic alterations in nasopharyngeal carcinoma: loss of heterozygosity and Epstein-Barr virus infection.

**DOI:** 10.1038/bjc.1997.460

**Published:** 1997

**Authors:** A. Mutirangura, C. Tanunyutthawongese, W. Pornthanakasem, V. Kerekhanjanarong, V. Sriuranpong, S. Yenrudi, P. Supiyaphun, N. Voravud

**Affiliations:** Department of Anatomy, Faculty of Medicine, Chulalongkorn University, Bangkok, Thailand.

## Abstract

**Images:**


British Journal of Cancer (1997) 76(6), 770–776
© 1997 Cancer Research Campaign

### Genomic alterations in nasopharyngeal carcinoma: loss of heterozygosity and Epstein–Barr virus infection

**A Mutirangura[1], C Tanunyutthawongese[2], W Pornthanakasem[1], V Kerekhanjanarong[3], V Sriuranpong[4], S Yenrudi[5], P Supiyaphun[3] and N Voravud[4]**

[1]Genetics Unit, Department of Anatomy, Faculty of Medicine, Chulalongkorn University, Bangkok 10330, Thailand; Departments of [2]Biochemistry and [3]Otolaryngology, Faculty of Medicine, Chulalongkorn University, Bangkok 10330, Thailand; [4]Medical Oncology Unit, Department of Medicine and [5]Department of Pathology, Faculty of Medicine, Chulalongkorn University, Bangkok 10330, Thailand

**Summary** Nasopharyngeal carcinoma is a subset of head and neck squamous cell cancers with unique endemic distribution and aetiological co-factors. Epstein–Barr virus has been revealed to be an important aetiological factor for most nasopharyngeal carcinomas. Nevertheless, additional genetic alterations may be involved in their development and progression. The aim of this study was to determine the likely chromosomal locations of tumour-suppressor genes related to Epstein–Barr virus-associated nasopharyngeal carcinoma. Fifty-six microsatellite polymorphic markers located on every autosomal arm were used to estimate the incidence of loss of heterozygosity in 27 Epstein–Barr virus-associated nasopharyngeal carcinomas. High frequencies of allelic loss were observed on chromosome 3p (75.0%) and 9p (87.0%). Chromosome 9q, 11q, 13q and 14q displayed loss in over 50%, while chromosome 3q, 6p, 16q, 19q and 22q exhibited loss in 35–50%. Furthermore, several other chromosomal arms demonstrated allelic loss in 20–35%. Additionally, 1 of the 27 cases showed microsatellite instability at multiple loci. These findings provide evidence of multiple genetic alterations during cancer development and clues for further studies of tumour-suppressor genes in Epstein–Barr virus-associated nasopharyngeal carcinoma.

**Keywords:** nasopharyngeal carcinoma; loss of heterozygosity; Epstein–Barr virus; allelotype; tumour-suppressor gene

Nasopharyngeal carcinoma (NPC) is a subset of head and neck squamous cell cancers (HNSCC) with unique endemic distribution and aetiological co-factors (Fandi et al, 1994). Although NPC is rare among Caucasians in Europe and North America, it is one of the most common cancers in southern China and among Eskimos in Arctic regions, where it has an incidence of 20–50 per 100 000 men. An intermediate incidence is noted in South-East Asia (Voravud, 1990). While HNSCC is closely associated with exposure to tobacco and alcohol, Epstein-Barr virus (EBV) appears to be an important aetiological factor for most NPC (Liebowitz, 1994).

Loss of function of tumour-suppressor genes has been implicated as being essential for solid tumour development and related to chromosomal rearrangement regarding the loss of normal chromosomes or segments (Knudson, 1971; Zhu et al, 1992). Various studies in NPC reported frequent allelic loss on chromosome 3p, 9p and 11q and homozygous deletion or hypermethylation of the *p16* gene (Huang et al, 1991; Choi et al, 1993; Lo et al, 1995–1996, Hui et al, 1996). The aim of this study was to investigate whether other tumour-suppressor genes are also involved in NPC development by analysing the loss of heterozygosity (LOH) on every autosomal arm. Interestingly, allelotyping of HNSCC have been well characterized (Nawroz et al, 1994; El-Naggar et al, 1995; Field et al, 1995). Allelic loss on chromosome 3p, 9p and 11q are also frequent events in HNSCC. In addition, frequent LOH was observed on other chromosomes, e.g. 6p, 8, 13q, 14q, 17p, 18q and 19q (Nawroz et al, 1994; El-Naggar et al, 1995; Field et al, 1995). It would be of great interest and importance to elucidate whether the genetic events in Epstein–Barr virus-associated NPC are similar or distinct from HNSCC.

*Received 6 December 1996*
*Revised 14 February 1997*
*Accepted 20 February 1997*

*Correspondence to:* A Mutirangura

### MATERIALS AND METHODS

### Tissues and DNA extraction

Primary NPC tissues were collected from 27 patients before treatment at Chulalongkorn University Hospital. The tissues were divided into two pieces. The first part was sent for routine histological examination. The second part was immediately stored in liquid nitrogen until further use. All the tumours were histologically ascertained to be undifferentiated NPC, according to the WHO classification. The 27 tumours included stages ranging from II to IV. Blood samples obtained by venipuncture from the same patients were used as constitutional controls. DNA was extracted from the tumour tissues and blood leucocytes by methods previously described (Maniatis et al, 1989).

### EBV detection and typing by PCR

For the detection and typing of EBV DNA in the tumour tissues, three previously described polymerase chain reaction (PCR) protocols were used with some modifications (Sample et al, 1990; Feinmesser et al, 1992; Lin et al, 1993). DNA from cell line B958, EBV-transformed human lymphocytes (American Type Culture Collection), was used as positive control and double-distilled water as negative control.

Duplex PCR was performed to detect EBV using two sets of primers. The first amplified the non-polymorphic EBV nuclear antigen 1 (EBNA-1), generating an approximately 610-bp DNA

**Figure 1** Autoradiographs showing PCR genotyping of EBV-infected NPC on a 2% agarose gel stained with ethidium bromide. The first lane from the left is φ174 *Hae* III standard DNA size marker. + and – are PCR products from positive controls, B958 cell line, and negative controls, double distilled water, respectively. Numbers indicate corresponding PCR products from NPC patients. (**A**) Duplex PCR generating 610-bp and 318-bp DNA fragments for EBNA-1 and human β-actin genomic sequence respectively. (**B**) PCR generating 246-bp and 153-bp DNA fragments for EBNA-3C of EBV type B and type A respectively. (**C**) PCR generating 184 bp and 168 bp DNA fragments for EBNA-2 of EBV type B and type A respectively fragment. The second amplified a human β-actin genomic sequence, generating an approximately 318-bp DNA fragment. The oligonucleotide sequences for both sets of PCR primers were identical to the ones previously reported (Feinmesser et al, 1992).

Two sets of PCR primers were used for EBV typing. The first primer amplified the EBV nuclear antigen 2 (EBNA-2), generating a DNA fragment of 168 bp for EBV type A and of 184 bp for EBV type B. The second one amplified the EBV nuclear antigen 3C (EBNA-3C), generating a DNA fragment of 153 bp for EBV type A and of 246 bp for EBV type B. The oligonucleotide sequences for both sets of PCR primers were identical to the ones previously reported (Sample et al, 1990; Lin et al, 1993).

The PCR reactions were performed in a total volume of 20 μl using 50 ng of the corresponding tumour DNA in 200 μM dNTP each, 1.5 mM magnesium chloride, 50 mM potassium chloride, 10 mM Tris-HCl (pH 9.0), 0.1% Triton X-100, 0.5 units of *Thermus aquaticus* DNA polymerase (Promega) and 0.5 μM of each primer. The PCR amplifications were performed as follows: initial denaturation at 94°C for 5 min, followed by 35 cycles of denaturation at 94°C for 30 s, annealing at 57°C for 30 s, with an extension at 72°C for 1 min and a final extension at 72°C for 7 min. The PCR products were then analysed using 2% agarose gel electrophoresis.

### Allelotyping

Fifty-six microsatellite markers for PCR analysis are listed in Table 1. For each chromosomal arm one to five markers were tested.

One strand of each primer pair was end labelled at 37°C for 1–2 h in a total volume of 10 μl containing 10 μM primer, 0.025 mCi [$\gamma$-$^{32}$P]ATP (Amersham) at 3000 Ci mmol$^{-1}$, 10 mM magnesium chloride, 5 mM DTT, 70 mM Tris-HCl (pH 7.6) and 10 units of T4 polynucleotide kinase (New England Laboratories). Without further separating of the unincorporated nucleotides, the kinase reaction was added to the PCR buffer mix.

The PCR reactions were performed in a total volume of 10 μl using 50 ng of genomic DNA in 200 μM dNTP each, 10 mM Tris-HCl (pH 8.4), 50 mM potassium chloride, 1.5 mM magnesium chloride (for all reactions, except NF1 and D20S470, 2.5 and 2.0 mM magnesium chloride, respectively, was added), 0.5 units of *Thermus aquaticus* DNA polymerase (Perkin Elmer Cetus) and the concentration was calculated from 0.05–0.5 μM of each primer.

The marker sets of (D3S1600, D3S966, D9S169), (D11S534, GABRB3, D9S51, D10S169), (GLUT2, D2S102, TCRD), (IL2RB, D8S88), (D16S287, D4S174), (D21S258, MFD133, D20S17, D1S103), (D12S341, D19S221) and (D2S131, D10S249) were analysed for LOH using multiplex PCR. The others were amplified as simplex PCR (Mutirangura et al, 1993).

Several PCR reactions, indicated in parentheses in Table 1, have been optimized for each primer set as follows: for reaction 1 and 3, the initial denaturation step at 95°C for 4 min, then followed by 25 cycles of denaturation at 94°C for 1 min, with 1 min of annealing at 55°C for reaction 1, or 52°C for reaction 3, extension at 72°C for 2 min and a final extension at 72°C for 7 min; for reaction 2, the initial denaturation step was 95°C for 4 min, then followed by

**Figure 2** Autoradiographs showing LOH analysis using microsatellite markers. Representative NPC tumours (T) and corresponding normal leucocytes (N) are shown with microsatellite markers indicated on the bottom. Markers D9S169, D11S897 and D13S284 reveal loss of upper alleles, and markers D3S1038 and TCRD reveal loss of lower alleles

© Cancer Research Campaign 1997

**Table 1** LOH and MSI data for each locus of 27 NPCs

| | Locus(C)[a] | Location | L/I(%) | 11 | 18 | 19 | 24 | 31 | 35 | 38 | 44 | 45 | 47 |
|---|---|---|---|---|---|---|---|---|---|---|---|---|---|
| Stage | | | | III | IV | IV | IV | R | IV | II | III | IV | IV |
| T | | | | 3 | 3 | 2 | 4 | | 4 | 2 | 2 | 1 | 4 |
| N | | | | 0 | 2[b] | 2[c] | 3 | | 3 | 0 | 1 | 2[b] | 2[b] |
| M | | | | 0 | 0 | 0 | 0 | | 1 | 0 | 0 | 0 | 0 |
| WHO | | | | II | II | II | III | II | III | II | II | II | III |
| EBV | | | | A | A | A | A | A | A | A | A | A | A |
| | D1S243 (1) | 1p36.1–2 | 7/24 (29.2) | – | – | U | – | + | – | + | – | U | – |
| | D1S103 (1) | 1q31q32 | 5/25 (20.0) | + | + | – | – | – | – | i | – | – | – |
| | D2S131 (3) | 2p | 2/20 (10.0) | – | U | – | – | – | – | i | – | – | – |
| | D2S102 (1) | 2q33q37 | 5/22 (22.7) | – | + | – | – | + | – | i | + | – | U |
| | D3S1038 (3) | 3p25 | 18/24 (75.0) | U | – | U | + | + | + | + | + | U | – |
| | D3S192 (3) | 3p25 | | U | – | U | + | N | N | + | + | – | – |
| | D3S966 (1) | 3p21.3 | 12/18 (66.6) | – | U | + | + | + | – | i | + | U | U |
| | D3S1600 (1) | 3p14 | 15/20 (75.0) | U | – | U | + | + | + | i | + | – | + |
| | GLUT2 (1) | 3q26.1–2 | 11/23 (47.8) | U | + | + | – | – | – | i | + | + | U |
| | D3S1744 (1) | 3q23q24 | | – | + | U | N | N | – | i | + | + | N |
| | D4S174 (1) | 4p11p15 | 6/23 (26.1) | – | – | – | – | – | – | i | + | U | – |
| | D4S1554 (2) | 4q11q35 | 4/23 (17.4) | – | U | + | N | – | + | i | – | – | – |
| | D5S392 (1) | 5p | 6/25 (24.0) | + | – | N | N | – | N | + | – | U | N |
| | D5S819 (2) | 5p | | U | U | – | N | U | – | – | U | U | – |
| | D5S82 (1) | 5q14q21 | 7/21 (33.3) | – | – | + | + | – | U | i | + | U | U |
| | D6S309 (2) | 6p | 10/21 (47.6) | + | – | + | + | – | U | i | + | – | + |
| | D6S503 (3) | 6q | 7/22 (31.8) | – | – | – | U | U | + | – | – | – | U |
| | D7S517 (1) | 7p | 4/24 (16.7) | – | + | – | – | – | – | + | + | – | – |
| | D7S486 (2) | 7q31 | 6/26 (23.1) | – | – | – | – | – | – | + | + | – | – |
| | NEFL (1) | 8p | 3/23 (13.0) | – | – | – | U | – | U | + | – | – | – |
| | D8S88 (1) | 8q22 | 3/22 (13.6) | – | – | – | U | – | – | i | – | – | U |
| | D9S169 (1) | 9p21 | 20/23 (87.0) | + | – | + | + | + | – | + | + | + | + |
| | IFNA (2) | 9p22 | | N | – | U | N | N | – | U | + | + | + |
| | D9S51 (1) | 9q | 11/22 (50.0) | – | + | + | – | + | U | + | – | – | + |
| | ABL1 (1) | 9q34 | | N | + | + | U | U | U | U | U | U | N |
| | D10S249 (3) | 10p | 4/21 (21.1) | – | – | – | N | – | – | – | – | – | + |
| | D10S169 (1) | 10q11.2 | 3/22 (13.6) | U | – | – | N | U | U | i | – | U | + |
| | D10S677 (1) | 10q | | – | N | N | N | – | – | – | – | – | N |
| | WT1 (1) | 11p13 | 7/25 (28.0) | U | U | + | N | – | – | + | U | + | – |
| | D11S554 (2) | 11p | | – | – | – | N | N | – | U | + | – | – |
| | D11S534 (1) | 11q13 | 7/27 (25.9) | U | – | – | N | – | U | i | – | – | U |
| | D11S956 (1) | 11q13 | | U | – | U | U | N | – | + | – | – | – |
| | INT2 (1) | 11q13.3 | | – | – | – | – | – | U | i | – | U | – |
| | D11S976 (1) | 11q23 | 14/26 (53.8) | U | – | U | N | N | + | + | + | + | – |
| | D11S897 (2) | 11q23 | | – | – | – | – | – | U | + | + | + | – |
| | D12S341 (2) | 12p | 7/23 (30.4) | – | – | – | + | + | – | i | U | – | – |
| | MFD133 (1) | 12q | 4/20 (20.0) | U | – | – | + | – | – | i | + | – | – |
| | D13S284 (2) | 13q14.2 | 14/22 (63.6) | N | + | + | + | + | – | i | + | – | + |
| | D13S119 (1) | 13q14.3-q22 | 6/20 (30.0) | – | U | + | N | – | U | – | + | U | U |
| | TCRD (1) | 14q11.2 | 10/19 (52.6) | – | U | U | U | + | U | U | U | + | – |
| | D14S118 (1) | 14q | 6/14 (42.9) | U | + | U | N | – | + | U | + | U | – |
| | GABRB3 (1) | 15q11q13 | 4/22 (18.2) | – | – | U | N | – | + | i | – | – | U |
| | D16S287 (1) | 16p13.11 | 4/22 (18.2) | – | U | – | + | + | – | – | – | – | – |
| | D16S511 (2) | 16q22q24 | 11/23 (47.8) | – | – | – | – | + | + | i | + | + | U |
| | D17S520 (1) | 17p12 | 8/27 (29.6) | – | – | – | – | – | – | i | – | – | – |
| | D17S1176 (2) | 17p | | – | – | – | – | – | – | + | – | – | – |
| | KRT9 (2) | 17q21 | 6/19 (31.6) | + | U | – | – | – | – | i | – | – | + |
| | D18S59 (1) | 18p11.2 | 0/20 (0) | U | – | – | – | – | – | – | – | – | – |
| | D18S35 (1) | 18q21.2 | 6/24 (25.0) | U | U | – | + | – | U | + | – | + | – |
| | DCC (1) | 18q21.1 | | U | – | U | + | – | + | i | U | – | – |
| | D19S221 (2) | 19p | 9/25 (36) | – | + | – | U | – | – | – | + | + | – |
| | D19S412 (2) | 19q | 2/19 (10.5) | U | – | – | U | U | – | i | – | U | – |
| | D20S470 (3) | 20p | 4/20 (20.0) | – | + | – | N | – | U | i | – | + | U |
| | D20S17 (1) | 20q12 | 3/20 (15.0) | – | – | U | + | – | – | U | + | – | – |
| | D21S258 (1) | 21q | 3/20 (15.0) | – | U | U | U | – | – | – | + | U | – |
| | IL2RB (1) | 22q | 10/22 (45.5) | U | – | + | N | + | – | + | – | + | – |

[a]C, PCR condition; L, number of positive LOH cases; I, number of informative cases; R, recurrence; A, EBV type A; B, EBV type B; +, positive LOH result; –, negative LOH result; i, microsatellite instability; U, uninformative result; N, not done.

© Cancer Research Campaign 1997

| 50 | 51 | 53 | 56 | 67 | 70 | 76 | 77 | 83 | 86 | 93 | 102 | 103 | 105 | 112 | 123 | 138 |
|---|---|---|---|---|---|---|---|---|---|---|---|---|---|---|---|---|
| IV | IV | III | IV | IV | IV | IV | IV | IV | III | IV | IV | IV | IV | IV | IV | III |
| 4 | 3 | 3 | 3 | 2 | 4 | 2 | 2 | 3 | 3 | 4 | 4 | 3 | 2 | 3 | 4 | 3 |
| 0 | 3 | 1 | 3 | 2[a] | 2[c] | 2[b] | 3 | 3 | 0 | 1 | 2[c] | 2[c] | 2[c] | 2[b] | 0 | 0 |
| 0 | 0 | 0 | 0 | 0 | 0 | 0 | 0 | 0 | 0 | 0 | 0 | 0 | 0 | 0 | 0 | 0 |
| II | III | II | II | II | III | III | III | II | II | III | III | III | III | II | III | II |
| A | A | A | A | A | A | A | A | B | A | A | A | A | A | A | A | A |
| − | + | − | + | − | − | − | − | − | − | U | + | + | + | − | − | − |
| U | + | + | + | − | − | − | − | − | − | − | − | − | − | − | − | − |
| U | − | − | + | U | − | − | − | − | U | − | + | U | U | − | − | − |
| − | + | U | − | − | − | − | − | U | − | + | − | − | U | − | − | − |
| − | U | + | + | U | + | + | + | + | + | + | U | + | + | U | U | U |
| − | + | + | + | U | + | U | U | + | + | + | + | U | U | + | − | − |
| + | + | + | + | − | − | − | U | + | U | − | + | + | U | + | U | N |
| + | − | + | + | + | − | − | + | + | + | + | + | + | U | U | U | N |
| U | U | U | U | U | + | − | U | − | + | + | + | − | − | + | − | U |
| − | + | N | − | N | N | N | + | − | U | + | + | − | − | + | − | − |
| + | + | + | + | − | − | U | − | + | − | − | U | − | − | − | − | − |
| − | + | U | − | − | − | − | − | − | − | − | − | + | − | − | − | − |
| U | − | N | − | − | N | + | − | + | − | U | − | + | − | − | − | − |
| − | − | − | − | U | − | U | U | N | U | − | + | + | − | − | − | N |
| U | + | − | − | + | − | − | − | − | − | − | + | + | − | U | − | − |
| − | + | − | − | U | + | − | + | U | U | + | + | − | U | − | − | − |
| − | − | + | U | − | − | − | + | + | − | U | − | + | − | + | + | − |
| − | − | + | − | − | U | − | − | − | U | − | − | U | − | − | − | − |
| − | − | + | U | − | + | − | − | + | − | + | − | − | − | − | − | − |
| − | − | + | − | U | − | − | − | − | U | − | + | − | − | − | − | − |
| − | − | − | − | + | − | − | − | U | + | − | − | − | + | − | U | − |
| + | − | + | U | + | U | U | U | N | U | + | + | + | U | U | U | + |
| + | − | + | + | + | U | + | + | + | U | U | + | + | U | + | U | + |
| U | − | − | + | + | − | U | U | − | − | U | U | U | U | U | U | U |
| U | − | + | U | N | U | U | U | U | − | U | − | U | U | U | + | + |
| − | + | + | − | − | − | U | − | N | − | U | U | + | U | − | − | − |
| U | U | U | U | − | U | U | − | + | + | − | U | U | U | U | U | U |
| − | − | N | − | N | − | N | − | + | + | − | U | − | − | N | − | _ |
| U | + | + | − | − | − | U | U | U | − | U | U | − | U | − | U | U |
| U | − | N | − | N | − | − | − | − | − | − | + | − | − | − | − | − |
| U | − | U | + | U | U | − | − | − | U | − | − | − | U | U | U | + |
| − | i | + | + | + | − | − | N | N | − | i | − | U | + | U | − | + |
| U | − | U | + | + | − | − | − | − | − | − | − | − | U | + | − | + |
| + | U | U | + | N | + | N | + | N | − | − | + | U | + | + | − | + |
| U | − | + | U | + | + | − | + | U | − | U | + | − | + | + | − | + |
| + | − | + | − | − | + | − | − | N | − | − | N | + | + | − | − | − |
| + | − | − | U | − | − | − | U | − | − | U | + | U | − | − | − | U |
| − | + | + | + | + | − | − | U | − | − | U | + | + | + | − | + | U |
| U | − | − | − | − | U | − | − | − | − | − | + | + | + | − | − | + |
| + | − | + | + | + | + | − | − | + | + | U | + | U | − | − | − | − |
| U | − | + | + | U | − | + | − | U | − | U | U | U | U | U | U | − |
| − | − | − | − | − | − | − | − | − | U | + | + | + | − | − | − | − |
| + | + | N | − | U | − | U | − | − | − | − | − | U | − | − | − | − |
| − | + | + | − | − | − | − | − | − | + | + | + | + | − | − | U | + |
| − | − | + | + | U | − | − | + | U | U | − | + | + | + | − | − | − |
| − | − | + | + | − | − | − | + | − | + | − | + | + | U | U | − | − |
| − | + | + | + | − | − | − | + | N | U | − | U | U | U | − | U | − |
| − | − | U | − | − | − | − | U | − |  | U | − | − | − | U | − | U |
| U | − | N | + | + | − | U | − | U | − | i | − | U | U | U | − | − |
| − | U | − | + | U | − | − | − | N | U | − | U | − | − | U | − | − |
| − | − | + | − | + | − | + | − | − | + | + | + | U | − | − | U | − |
| U | + | − | − | − | + | − | − | − | − | − | − | − | U | − | − | U |
| U | − | + | − | − | − | U | − | − | − | − | − | + | U | − | − | − |
| U | − | + | − | U | − | − | U | − | − | U | − | − | − | − | U | − |
| − | − | + | − | U | + | − | − | U | − | − | − | − | U | − | − | − |
| − | + | + | + | + | + | − | − | U | U | + | − | − | − | − | U | − |

© Cancer Research Campaign 1997

**Figure 3** Frequency of allelic loss for autosome in NPC. Allelotyping was accomplished using polymorphic microsatellite analysis. The probes used are listed in Table 1

**Figure 4** Autoradiograph showing the microsatellite instability at KRT9 and DCC loci. N, normal DNA; T, tumour DNA

five cycles of step-down PCR denaturation at 94°C for 1 min, with 1 min of annealing at 60°C, 59°C, 58°C, 57°C and 56°C, extension at 72°C for 2 min and 25 cycles of denaturation at 94°C for 1 min, annealing at 55°C for 1 min, with extension at 72°C for 2 min and a final extension at 72°C for 7 min.

Two microlitres of each reaction were mixed with 1 µl of formamide-loading buffer, heated at 95°C for 2 min, put on ice for 30 s and then loaded onto 6% polyacrylamide/7 M urea gel. DNA fragments were size fractionated at 70 W until the tracking dye reached the appropriate point on the gel. After electrophoresis, the wet gel was transferred to filter paper (Watman), wrapped with Saran wrap and exposed to Kodak T-mat radiographic film for 6–24 h at –70°C with an intensifying screen.

### RESULTS

Twenty-seven EBV-associated NPC samples were selected for LOH studies. All biopsied specimens were histologically confirmed. Among these 27 cases, 15 were WHO type II and the others were WHO type III. Twenty-six cases were infected with EBV subtype A and one case with type B (Figure 1 and Table 1).

### LOH in NPC

A panel of 56 microsatellite polymorphic markers representing every chromosomal arm was used to screen for LOH frequency. Table 1 shows the polymorphic loci used to test each chromosomal arm, patient staging, EBV typing and LOH, as well as microsatellite instability (MSI) results. Results representative for LOH are shown in Figure 2. Frequencies of LOH for each autosomal arm are represented in Figure 3.

The frequencies of LOH from each chromosomal arm varied from 0% to 87%. Chromosome 3p and 9p with 78% and 87%, respectively, revealed higher incidence than other chromosomal arms. For chromosome 3p, further analysis displayed that there were at least two LOH loci, which were 3p14, D3S1600 and 3p25, D3S192 and D3S1038. Two cases, 51 and 70, showed LOH from 3p25 but not 3p14. In contrast, two cases, 47 and 50, revealed loss from 3p14 but not 3p25. Two cases, 35 and 93, presented loss from both 3p14 and 3p25 but not 3p21. Thus, 3p14 and 3p25 were two separate LOH loci. Other regions with allelic loss over 50% were on chromosome 9q (50.0%), 11q (53.8%), 13q (63.6%) and 14q (52.6%). Further analyses of chromosome 11q revealed higher frequency of LOH on 11q23 (53.8%), while only 7 out of 27 (25.9%) had LOH on 11q13. Additional analyses on chromosome 13 displayed that the incidence of LOH for D13S284, located on 13q14, was higher than D13S119, located on 13q14.3-q22. LOH between 35% and 50% was noted on chromosome 3q (47.8%), 16q (47.8%), 19p (36.0%) and 22q (45.5%). Finally, several other chromosomes demonstrated allelic loss in 20–35%.

### MSI in NPC

Out of 27 samples tested, MSI for multiple loci was presented in only one case, i.e. 38. MSI was revealed in 25 of 56 loci. In addition, sample 93 demonstrated MSI on two loci, D11S956 and D18S35, and sample 53 showed MSI on one locus, D11S956. Representative results for MSI are shown in Figure 4. No significant clinical difference was noted regarding these cases.

### DISCUSSION

Several areas of chromosomal loss during cancer development and progression are associated with inactivation of both tumour-suppressor gene alleles (Huang et al, 1991; Zhu et al, 1992; Nawroz et al, 1994). In addition, they are correlated with the histopathology, staging and clinical outcome of cancer (Broder et al, 1995). Here we demonstrated several chromosomes with significant LOH in

 © Cancer Research Campaign 1997

NPC. While several LOH loci are common to HNSCC, some appear to be unique to NPC progression.

Previously Choi et al (1993) showed LOH on chromosome 3p for all of the informative 35 NPC cases and Huang et al (1994) studied chromosome 9p and found allelic loss on 11 samples from 18 NPC. Consistently, in this study, two of the highest incidences of allelic loss have been shown on chromosomes 3p (78%) and 9p (87%). Three LOH loci have been reported on chromosomes 3p, 3p14, 3p21 and 3p25 (Maestro et al, 1993). This study revealed that, at least, the 3p14 and 3p25 loci are associated with NPC development. It should also be noted that a putative tumour-suppressor gene, *VHL*, is the candidate gene on chromosome 3p25 (Latif et al, 1993). For 9p, the LOH locus has been defined on chromosome band 9p21; and gene *p16*, which controls cell cycles, has previously been shown to have homozygous deletion or hypermethylation (Lo et al, 1995, 1996). Interestingly, 9p LOH has now been well documented in precancerous lesions of HNSCC (El-Naggar et al, 1995). It is interesting to investigate whether genetic alterations of chromosome 9p might also be an early event of NPC carcinogenesis.

Allelic loss of chromosome 11q has been observed in several other tumour types, such as HNSCC, breast, ovary and lung (Nawroz et al, 1994; Gudmundsson et al, 1995*a*; Iizuka et al, 1995). Poor prognosis of breast and ovarian cancer has been associated with 11qLOH (Gudmundsson et al, 1995*a*). At least three LOH loci have been delineated on chromosome 11q, 11q13, 11q15 and 11q23 (Iizuka et al, 1995). In addition, 11q13 is a chromosome region with a high frequency of amplification in HNSCC (Meredith et al, 1995). This results in allelic imbalance and may be difficult to distinguish from LOH. This study described a higher frequency of allelic loss in NPC on 11q23 than on 11q13. It should also be noted that the LOH on 11q23 may be related to the ataxia-telangiectasia locus (Savitsky et al, 1995).

Chromosome 13qLOH is also frequently detected in several cancers, such as retinoblastoma, breast cancer and HNSCC (Zhu et al, 1992; Nawroz et al, 1994; Gudmundsson et al, 1995*b*). This study has shown that the common LOH locus in NPC may be located proximal to 13q14.3. At least two tumour-suppressor genes are located proximal to this region. The first is *Rb*, retinoblastoma gene, and the other is *BRCA2*, the candidate gene for the second locus of the familial breast cancer syndrome (Zhu et al, 1992; Gudmundsson et al, 1995*b*). Previous mutation analysis of *Rb* on NPC demonstrated negative results (Sun et al, 1993). Thus, it is tempting to hypothesize that the *BRCA2* tumour-suppressor gene may be responsible for NPC development.

The allelic loss of chromosome 14q was also frequently found in other types of cancer, e.g. bladder cancer, neuroblastoma, colorectal cancer and HNSCC (Fong et al, 1992; Young et al, 1993; Nawroz et al, 1994; Chang et al, 1995). A recent study has delineated two tumour-suppressor gene loci on chromosome 14, i.e. 14q12 and 14q32 (Chang et al, 1995). Interestingly, LOH of 14q is associated with an advanced phenotype of neuroblastoma and frequently found in advanced colorectal cancer (Fong et al, 1992; Young et al, 1993).

Chromosome 17p is one of the most common regions with genetic alterations reported in cancer. *p53*, the best known tumour-suppressor gene, is located on this chromosome (Carson et al, 1995). *p53* alterations, including protein expression and mutations, are common in HNSCC while mutation of *p53* in NPC is infrequent (Field et al, 1991; Boyle et al, 1993; Shin et al, 1994; Brennan et al, 1995; Chakrani et al, 1995). The LOH study of this chromosome supported the mutation data in which allelic loss of chromosome 17p was noticed in only 30% of the NPC, while previous reports revealed 50% LOH of the HNSCC (Nawroz et al, 1994; Field et al, 1995).

Among 27 cases tested, 15 were WHO type II and 12 were WHO type III. The average frequencies of LOH for each chromosome region were 0.34 and 0.31 for WHO type II and III respectively. Interestingly, LOH was found to be more frequent on chromosome 4p, 7p, 9q, 11q and 22q for WHO type II, while a higher frequency of LOH for WHO type III was reported on chromosome 6p and 15q. However, because of the limited number of tumours, these comparative data are not statistically significant.

MSI is presented as variations in the length of microsatellite repeats in tumour DNA when compared with matched normal DNA. The abnormality in the size of the microsatellite loci has been observed in various types of cancer as well as in hereditary non-polyposis colorectal cancer (HNPCC) (Thibodeau et al, 1993). In HNPCC, mutations in a number of DNA mismatch repair genes (*hMSH2*, *hMLH1*, *hPMS1* and *hPMS2*) have been reported. Thus, MSI may be the consequence of decreased accuracy of the DNA mismatch repair system during DNA replication, which might facilitate the accumulation of mutations (Rhyu, 1996). This study has presented MSI of multiple loci in only one out of 27 NPC samples tested. This suggests that the phenomenon of MSI is a relatively rare event during NPC development.

NPC is a unique subclassification of HNSCC as a result of its endemic distribution and aetiological cofactors. It would be of great interest and importance to elucidate whether the genetic events in Epstein–Barr virus-associated NPC are similar or distinct from HNSCC. Previous HNSCC studies have demonstrated a high frequency of LOH on chromosomes 3p, 3q, 6p, 8p, 8q, 9p, 11q, 13q, 14q, 17p, 18q and 19q (Nawroz et al, 1994; El-Naggar et al, 1995; Field et al, 1995). Nawroz et al (1994) studied 29 HNSCCs and showed 67, 50, 38, 40, 38, 72, 61, 54, 39, 52, 23 and 40% allelic losses respectively. Additionally, Field et al (1995) tested 80 specimens and found LOH more frequently on chromosome 3p, 8p, 9p, 13q, 17p, 18q and 19q for 52, 35, 62, 27, 50, 49 and 29% respectively. Finally, El-Naggar et al (1995) studied 20 patients for LOH on chromosome 3p, 5q, 8p, 9p, 9q, 11q and 17p, and a high incidence of LOH in invasive carcinoma was observed at 9p (72%), 8p (53%), 3p (47%), 9q (35%) and 11q (33%). Similar incidences on chromosomes 3p (78%), 3q (48%), 6p (48%), 9p (87%), 11q (54%), 13q (64%) and 14q (43%) have also been detected regarding NPC. However, NPC revealed lower incidences of LOH on chromosomes 8p (13%), 17p (30%) and 19q (11%). In contrast, this study has shown frequent allelic loss regarding NPC on chromosomes 16q (48%) and 22q (46%). As several genetic alterations of NPC and HNSCC are similar, the multistep processes for the development and progression of both cancers overlap. However, some genetic changes seem to be unique in the biology of NPC development.

It would also be of interest to compare these allelotyping data with the allelic loss pattern of other EBV-associated neoplasias, for example post-immunosuppression/transplant, AIDS-related and Burkitt's lymphomas. However, there is only limited knowledge of LOH for comparison at present.

### ACKNOWLEDGEMENTS

The authors are deeply indebted to the staff of the Department of Otolaryngology and the Radiotherapy Section, Department of

© Cancer Research Campaign 1997 

Radiology, Chulalongkorn University Hospital, for the recruitment of patients and collection of materials. We also thank Dr David H Ledbetter and Dr Lisa G Shaeffer for providing some STR primers, Mrs Bungon Changchup and Mr Anucha Karnthaworn for technical assistance and Dr Henry Wilde, Ms Petra Hirsch, Dr Dong M Shin and Dr Karol Sikora for their critical review of the manuscript. This work was supported by the Rachadapisek Sompoj China Medical Board research fund, Molecular Biology Project, Faculty of Medicine, Chulalongkorn University and the Thailand Research Fund.

### REFERENCES

Brennan JA, Mao L, Hruban RH, Boyle JO, Eby YJ, Koch WM, Goodman SN and Sidransky D (1995) Molecular assessment of histopathological staging in squamous-cell carcinoma of the head and neck. *N Engl J Med* **332**: 429–435

Broder S and Karp JE (1995) Progress against cancer (Review). *J. Cancer Res Clin Oncol* **121**: 633–647

Boyle JO, Hakim J, Koch W, van der Riet P, Hruban RH, Roa RA, Correo R, Eby YJ, Ruppert JM and Sidransky D (1993) The incidence of p53 mutations increases with progression of head and neck. *Cancer Res* **53**: 4477–4480

Carson DA and Lois A (1995) Cancer progression and p53 (Review). *Lancet* **346**: 1009–1011

Chakrani F, Armand J-P, Lenoir G, Ju L, Liang J-P, May E and May P (1995) Mutations clustered in exon 5 of the p53 gene in primary nasopharyngeal carcinomas from southeastern Asia. *Int J Cancer* **61**: 316–320

Chang WY, Cairns P, Schoenberg MP, Polascik TJ and Sidransky D (1995) Novel suppressor loci on chromosome 14q in primary bladder cancer. *Cancer Res* **55**: 3246–3249

Choi PHK, Suen MWM, Huang DP, Lo K-W and Lee JCK (1993) Nasopharyngeal carcinoma: genetic changes, Epstein-Barr virus infection, or both. *Cancer* **15**: 2873–2878

El-Naggar AK, Hurr K, Batsakis JG, Luna MA, Goepfert H and Huff V (1995) Sequential loss of heterozygosity at microsatellite motifs in preinvasive and invasive head and neck squamous carcinoma. *Cancer Res* **55**: 2656–2659

Fandi A, Altun M, Azli N, Armand JP and Cvitkovic E (1994) Nasopharyngeal cancer: epidemiology, staging and treatment. *Semin Oncol* **21**: 382–397

Feinmesser R, Miyazaki I, Cheung R, Freeman JL, Noyek AM and Dosch H-M (1992) Diagnosis of nasopharyngeal carcinoma by DNA amplification of tissue obtained by fine-needle aspiration. *N Engl J Med* **326**: 17–21

Field JK, Spandidos DA, Malliri A, Gosney JR, Yiagnisis M and Stell PM (1991) Elevated p53 expression correlates with a history of heavy smoking in squamous cell carcinoma of the head and neck. *Br J Cancer* **64**: 573–577

Field JK, Kiaris H, Risk JM, Tsiriyotis C, Adamson R, Zoumpourlis V, Rowley H, Taylor K, Whittaker J, Howard P, Beirne JC, Gosney JR, Woolgar J, Vaughan ED, Spandidos DA and Jones AS (1995) Allelotype of squamous cell carcinoma of the head and neck: fractional allele loss correlates with survival. *Br J Cancer* **72**: 1180–1188

Fong CT, White PS, Peterson K, Sapienza C, Cavenee WK, Kern SE, Vogelstein B, Cantor AB, Look AT and Brodeur GM (1992) Loss of heterozygosity for chromosomes 1 or 14 defines subsets of advanced neuroblastomas. *Cancer Res* **52**: 1780–1785

Gudmundsson J, Barkardottir RB, Eiriksdottir G, Baldursson T, Arason A, Egilsson V and Ingvarsson S (1995*a*) Loss of heterozygosity at chromosome 11 in breast cancer: association of prognostic factors with genetic alterations. *Br J Cancer* **72**: 696–701

Gudmundsson J, Jòhannesdottir G, Bergthorsson JT, Arason A, Ingvarsson S, Egilsson V and Barkardottir RB (1995*b*) Different tumor types from BRCA2 carriers show wild-type chromosome deletions on 13q12–q13. *Cancer Res* **55**: 4830–4832

Huang DP, Lo KW, Choi PH, Ng AY, Yiu GK and Lee JC (1991) Loss of heterozygosity on the short arm of chromosome 3 in nasopharyngeal carcinoma. *Cancer Genetics Cytogenetics* **54**: 91–99

Huang DP, Lo K-W, Van Hasselt A, Woo JKS, Choi PHK, Leung S-F, Cheung S-T, Cairns P, Sidransky D and Lee JCK (1994) A region of homozygous deletion on chromosome 9p21-22 in primary nasopharyngeal carcinoma. *Cancer Res* **54**: 4003–4006

Hui ABY, Lo K-W, Leung S-F, Choi PHK, Fong Y, Lee JCK and Huang DP (1996) Loss of heterozygosity on the long arm of chromosome 11 in nasopharyngeal carcinoma. *Cancer Res* **56**: 3225–3229

Iizuka M, Sugiyama Y, Shiraishi M, Jones C and Sekiya T (1995) Allelic losses in human chromosome 11 in lung cancers. *Genes Chromosomes Cancer* **13**: 40–46

Knudson AG JR (1971) Mutation and cancer: statistical study of retinoblastoma. *Proc Natl Acad Sci USA* **68**: 820

Latif F, Tory K, Gnarra J, Yao M, Duh FM, Orcutt ML, Stackhouse T, Kuzmin I, Modi W, Geil L, Schmidt L, Zhou F, Li H, Wei MH, Chen F, Glenn G, Choyke P, Walther MM, Weng Y, Duan D-SR, Dean M, Glavac D, Richards FM, Crossey PA, Ferguson-Smith MA, Le Paslier D, Chumakov I, Cohen D, Chinault AC, Maher ER, Linehan WM, Zbar B and Lerman M (1993) Identification of the von Hippel-Lindau disease tumor suppressor gene. *Science* **260**: 1317–1320

Liebowitz D (1994) Nasopharyngeal carcinoma: the Epstein-Barr virus association. *Semin Oncol* **21**: 376–381

Lin J-C, Lin S-C, De BK, Chan W-P and Evatt BL (1993) Precision of genotyping of Epstein-Barr virus by polymerase chain reaction using three gene loci (EBNA-2, EBNA-3C, and EBER): predominance of type A virus associated with Hodgkin's disease. *Blood* **81**: 3372–3381

Lo K-W, Huang DP and Lau KM (1995) P16 gene alterations in nasopharyngeal carcinoma. *Cancer Res* **55**: 2039–2043

Lo K-W, Cheung S-T, Leung S-F, Van Hasselt A, Tsang Y-S, Mak K-F, Chung Y-F, Woo JKS, Lee JCK and Huang DP (1996) Hypermethylation of the p16 gene in nasopharyngeal carcinoma. *Cancer Res* **56**: 2721–2725

Maestro R, Gasparotto D, Vuksavljevic T, Barzan L, Sulfaro S and Boiocchi M (1993) Three discrete regions of deletion in head and neck cancers. *Cancer Res* **53**: 5775–5779

Maniatis T, Fritsch EF and Sambrook J (1989) *Molecular Cloning: A Laboratory Manual*, 2nd edn. Cold Spring Harbor Laboratory: Cold Spring Harbor, NY

Meredith SD, Levine PA, Burns JA, Gaffey MJ, Boyd JC, Weiss LM, Erickson NL and Williams ME (1995) Chromosome 11q13 amplification in head and neck squamous cell carcinoma. Association with poor prognosis. *Archives of Otolaryngology – Head & Neck Surgery* **121**: 790–794

Mutirangura A, Greenberg F, Butler MG, Malcolm S, Nicholls RD, Chakravarti A and Ledbetter DH (1993) Multiplex PCR of three dinucleotide repeats in the Prader-Willi/Angelman critical region (15q11–q13): molecular diagnosis and mechanism of uniparental disomy. *Hum Molec Genet* **2**: 143–151

Nawroz H, Van Der Riet P, Hruban RH, Koch W, Ruppert JM and Sidransky D (1994) Allelotype of head and neck squamous cell carcinoma. *Cancer Res* **54**: 1152–1155

Rhyu MS (1996) Molecular mechanisms underlying hereditary nonpolyposis colorectal carcinoma. *J Natl Cancer Inst* **88**: 240–251

Sample J, Young L, Martin B, Chatman T, Kieff E, Rickinson A and Kieff E (1990) Epstein-Barr virus type 1 and 2 differ in their EBNA-3A, EBNA-3B, and EBNA-3C genes. *J Virol* **64**: 4084–4092

Savitsky K, Bar-Shira A, Gilad S, Rotman G, Ziv Y, Vanagaite L, Tagle DA, Smith S, Uziel T, Sfez S, Ashkenazi M, Pecker I, Frydman M, Harnik R, Patanjali SR, Gatti RA, Chessa L, Sanal O, Lavin MF, Miki T, Weissman SM, Lovett M, Collin FS and Shiloh YA (1995) Single ataxia telangiectasia gene with a product similar to PI-3 kinase. *Science* **268**: 1749–1753

Shin DM, Kim J, Ro JY, Hittelman J, Roth JA, Hong WK and Hittelman WN (1994) Activation of p53 gene expression in premalignant lesions during head and neck tumorigenesis. *Cancer Res* **54**: 321–326

Sun Y, Hegamyer G and Colburn NH (1993) Nasopharyngeal carcinoma shows no detectable retinoblastoma susceptibility gene alterations. *Oncogene* **8**: 791–795

Thibodeau SN, Bren G and Schaid D (1993) Microsatellite instability in cancer of the proximal colon. *Science* **260**: 816–819

Voravud N (1990) Cancer in the Far East. In *Treatment of Cancer*, 2nd edn, Sikora K and Halman KE. (eds) pp. 887–894. Chapman and Hall Medical: London

Young J, Leggett B, Ward M, Thomas L, Buttenshaw R, Searle J and Chenevix-Trench G (1993) Frequent loss of heterozygosity on chromosome 14 occurs in advanced colorectal carcinomas. *Oncogene* **8**: 671–675

Zhu X, Dunn JM, Goddard AD, Squire JA, Becker A, Phillips RA and Gallie BL (1992) Mechanisms of loss of heterozygosity in retinoblastoma. *Cytogenet Cell Genet* **59**: 248–252

© Cancer Research Campaign 1997